# Lipid Membrane Nanosensors for Environmental Monitoring: The Art, the Opportunities, and the Challenges

**DOI:** 10.3390/s18010284

**Published:** 2018-01-18

**Authors:** Georgia-Paraskevi Nikoleli, Dimitrios Nikolelis, Christina G. Siontorou, Stephanos Karapetis

**Affiliations:** 1 Laboratory of Inorganic & Analytical Chemistry, School of Chemical Engineering, Dept 1, Chemical Sciences, National Technical University of Athens, 157 80 Athens, Greece; Dimitrios.Nikolelis@chem.uoa.gr (G.-P.N.); stevekara@chem.uoa.gr (S.K.); 2 Laboratory of Environmental Chemistry, Department of Chemistry, University of Athens, 157 72 Athens, Greece; 3 Laboratory of Simulation of Industrial Processes, Department of Industrial Management and Technology, School of Maritime and Industry, University of Piraeus, 185 34 Piraeus, Greece; csiontor@unipi.gr

**Keywords:** lipid films, nanosensors, environmental monitoring

## Abstract

The advent of nanotechnology has brought along new materials, techniques, and concepts, readily adaptable to lipid membrane-based biosensing. The transition from micro-sensors to nano-sensors is neither straightforward nor effortless, yet it leads to devices with superior analytical characteristics: ultra-low detectability, small sample volumes, better capabilities for integration, and more available bioelements and processes. Environmental monitoring remains a complicated field dealing with a large variety of pollutants, several decomposition products, or secondary chemicals produced ad hoc in the short- or medium term, many sub-systems affected variously, and many processes largely unknown. The new generation of lipid membranes, i.e., nanosensors, has the potential for developing monitors with site-specific analytical performance and operational stability, as well as analyte-tailored types of responses. This review presents the state-of-the art, the opportunities for niche applicability, and the challenges that lie ahead.

## 1. Introduction

Biosensors are analytical devices that transform chemical information contained within a sample into an analytically useful signal; this information can range from the concentration of a specific sample component to total composition analysis. Biosensors contain two basic components connected in series: a recognition element and a physicochemical transducer. The recognition element is of biological nature (“receptor”, enzyme, antibody, natural receptor, cell, etc.) and it is retained in direct contact with the transduction component. The target analyte interacts with the recognition element in a way similar to interactions occurring in natural systems. The biochemical information is transformed into an electrical, optical, piezoelectric, etc., output by the transducer. The resultant device has a relatively small size and can be made portable. The possibility of using natural chemoreception in handheld detectors is intriguing indeed, especially in the promise of achieving the selectivity and sensitivity of these processes. In that sense, biosensors are ecological-relevant and quite suitable for environmental analysis.

Research on environmental biosensing remains blooming for two decades now, attracting scientists from diverse fields. From an analytical viewpoint, biosensors offer a number of benefits when compared to the conventional techniques (e.g., chromatography or immunoassays), including minimal sample preparation, real time detection, rapid response times, portability, etc. Some biosensors for biological oxygen demand (BOD) measurements are available commercially and, despite their higher cost [1], they are generally preferred over the BOD kits mostly due to the time of analysis: biosensors require 60 min to give a response whereas the kits need five days. Other commercial devices include detectors for nitrates, dioxin, and dioxin-like compounds (for a recent review on commercial devices, see [2]). Biosensor technology is very versatile and readily amenable to intended use customizations. Some biosensor platforms, called multiple-use, have the ability to be repeatedly calibrated and can monitor both, the increase and decrease of the analyte concentrations; this technology can be proven very useful in remediation engineering, where the monitoring of the decrease (or the rate of decrease) of the level of a pollutant is required for planning further activities. On the other hand, single-use devices, i.e., sensors that cannot be rapidly and reproducibly regenerated for a second analysis, could be used for rapid quality assessments.

Nanotechnology is playing an increasingly important role for the development of nanobiosensors. The use of nanomaterials has allowed the introduction of many new signal transduction technologies in biosensors and, also, improved their sensitivity and performance. Due to their submicron size, nanosensors and nanoprobes are revolutionizing the field of chemical analysis and enable rapid analysis of multiple substances for in the field detection of food toxicants and environmental pollutants. Recent progress in nanotechnology has provided the opportunity to mass produce affordable devices and to integrate them into marketed systems for environmental monitoring or the detection of food toxicants. These devices can detect a broad range of chemical and micro-biological toxicants, such as toxins, insecticides, pesticides, herbicides, microorganisms, bacteria, viruses, and other microorganisms, polycyclic aromatic hydrocarbons (PAHs), hydrazines, phenolic compounds, allergens, genetically-modified foods, hormones, dioxins, etc. Artificial sensing mimicking natural chemoreception has just started to emerge, based on the effective coupling of complex biomolecular function units with electronics [3,4]. The use of gustatory or olfactory receptor-based biosensors gave rise to bioelectronic tongues and noses, respectively, with advanced performance in food quality [5] or medical diagnostics [6]. It is generally acknowledged that lipid membranes play an important role in natural sensing [7], although the full scale of their functionality is not easy to grasp.

Lipids are amphiphilic molecules, which possess both hydrophilic head groups and hydrophobic chains. Since the discovery of black lipid films by Mueller et al. [8], there have been various attempts to use model bilayer lipid membranes (BLMs), solventless and free standing, for biosensor construction and for other applications. Although the initial drive for the implementation of lipid bilayers in biosensors has been undoubtedly the reconstitution of natural membranes in vitro [9], without much knowledge on their functionality or how much of that functionality could be actually exploited, the studies conducted managed to reveal a multifaceted role in both, device engineering and signal production. The most obvious benefit derives from the nature-like environment that the membranes provide for the immobilization of proteins [10]. Further, the dynamics of the bilayer in physical terms and the way that the interaction between the biological moiety and the analyte impacts this meta-stable system establish a generic signal amplification mechanism [11]. As regards construction, the tendency of lipids to assemble spontaneously into a bilayer under simple artificial conditions allows the formation of model membranes using relatively simple laboratory setups [12].

**Figure 1:**
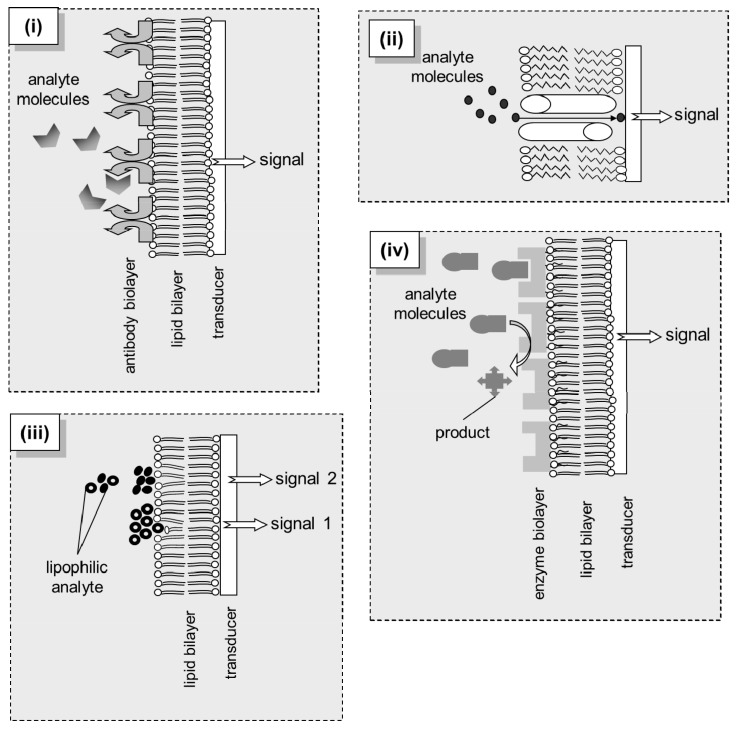
Different principles of biosensing via structured lipid bilayer interfaces: (**i**) Immunosensing: the antigen is recognized by the antibody attached to the lipid film; complementation induces structural changes to the immobilized unit that trigger transient modifications to the packing of lipids, expressed as a transient modification of the transmembrane current. (**ii**) Channel-based sensing: transport or channel proteins are incorporated into the lipid bilayer; these moieties transfer the analyte through the membrane, thereby permanently increasing the transmembrane current. (**iii**) Lipid adsorption-based sensing: mixtures of similar lipophilic compounds are adsorbed onto the bilayer surface inducing lipid packing modifications that result in transmembrane current alterations; using a slight difference at the partition coefficients of the mixture components, a series of discrete signals can be produced, each one indicating, with adequate resolution, each analyte species in the mixture. (**iv**) Enzyme-based sensing: the analyte is converted by the membrane-immobilized enzymes; the reaction induces electrochemical changes to the bilayer-solution interface (Debye length) resulting in modifications of surface charge or dipolar potential of the membrane, expressed as a transient modification of the transmembrane current.

However, these free standing BLMs were fragile and, thus, unsuitable for long-term use. Their low mechanical and electrical stability was the main obstacle to their practical applications. Recent advances in the stabilization of lipid bilayers have resulted in preparing lipid film based biosensors for the detection of a large variety of compounds in real samples using a variety of detection strategies (Figure 1). Lipid membranes represent an appropriate biocompatible structure for the development of novel biosensors with rapid response times (on the order of a few seconds) and high sensitivity (i.e., nanomolar detection limits) that may eventually be used for environmental monitoring applications. Most of these biosensors are cost efficient, easy-to-use, fast-responding, and portable [9]. They could be appropriate alternatives to the expensive, bulky, time-consuming standard analytical methods (e.g., chromatographic techniques). The new generation of lipid membrane nanosensors has the potential for developing monitors with site-specific operational stability and analyte-tailored type of response.

The bilayer lipid membrane has been mostly coupled to electrochemical transducers and engaged a variety of sensing strategies, including ion sensing, material transport, electric excitability, gated channels, antigen-antibody binding, phase shifting and conformational re-arrangements (Figure 1). The lipid-based platforms have been proven very versatile and prone to site-specific, analyte-specific or sample-specific customizations. Ultra-thin lipid films make the use of even highly expensive moieties, such as engineered receptors or DNA, to become economically possible [13]. Detection requires the molecules of the analyte to diffuse into and react with the biological system; the sensitivity and selectivity of the sensor depend strongly upon the biological moiety and its affinity for the target [10], while kinetics can be regulated by the surrounding micro-environment (pH, ionic strength, temperature, etc.) [14]. The reaction *per se* and/or the result of the reaction change the bilayer in a variety of ways: alterations of the dipolar potential, the molecular packing and fluidity or the surface charge density have been commonly associated with the mechanism of signal generation [9]. These perturbations in the physical state of the bilayer result in minor or major alterations of the transmembrane potential, manifested as ion current increases or decreases, the magnitude of which relates to the degree and extend of the transmembrane potential alteration and, in effect, to the concentration of the analyte in the sample [9]. Depending on the nature of the biological interaction and its impact on the bilayer, the sensor’s response could be transient assuring its reversibility; otherwise, an external intervention would be necessary for regenerating the sensor [15].

This work reviews various nanostructure lipid film based nanobiosensors. The chapter provides the state of art of design and microfabrication of prototype lipid membrane nanosensing devices. The engineering principles for achieving rapid in the field detection are also presented, whereas the challenges that lie ahead are thoroughly discussed.

## 2. Methods for Preparing Biosensors Based on Lipid Films

Over the last two decades, a number of techniques have been reported in the scientific literature for the preparation of stabilized lipid membranes that are not prone to electrical or mechanical failure and are, thus, suitable for practical applications. Most of these techniques, described below, yield lipid films with length less than 1 µm that fit the connotation of nanosensors. When coupled to electrochemical transduction, the nano dimensions can be retained. Electrochemical biosensors have been widely studied mainly due to a well-established low-cost infrastructure, readily amenable to miniaturization.

Stabilized polymerized lipid films can be constructed within the pores of a filter paper or on carbon paste electrodes. Both architectures have a microscale structure.

### 2.1. Metal Supported Lipid Layers

**Figure 2:**
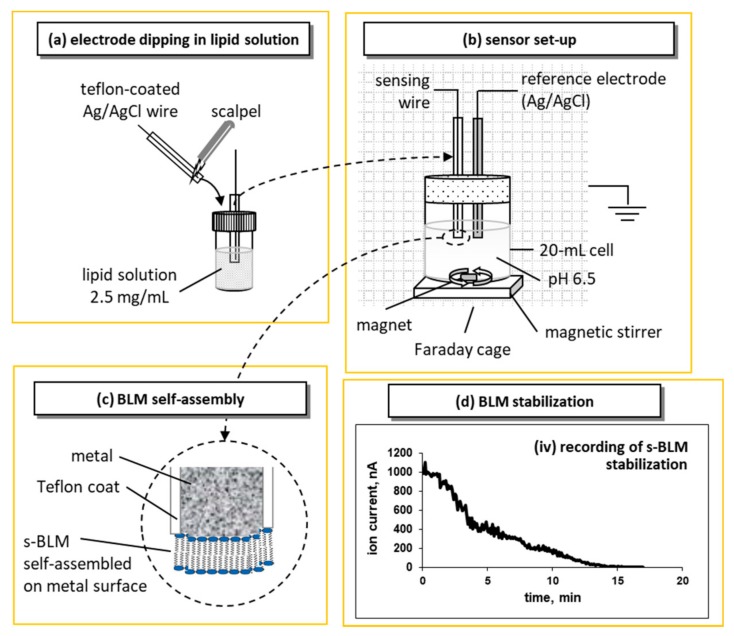
Schematic of the sensor, measurement setup, and lipid self-assembly process (not drawn to scale): (**a**) the sensing electrode is tipped with a scalpel and immediately immersed in lipid solution before dipped in the electrolyte solution. (**b**) The electrochemical setup consists of a 20-mL cell and a two-electrode configuration, i.e., the sensing electrode and a Ag/AgCl reference electrode, placed in a grounded Faraday cage; an external DC potential of 25 mV is applied between the electrodes and the ionic current through the BLM is measured with a digital electrometer; the cell is stirred using a magnetic stirrer. (**c**) Upon immersion, the lipid droplet attached to the wire is self-assembled into a bilayer that has one layer adsorbed on the metal surface and the other facing the aqueous solution. (**d**) Recording of the ion current decrease during the self-assembly process; recording started at the immersion of the sensing electrode in the electrolyte solution.

A simple and reproducible method for preparing a stabilized bilayer lipid membrane (sBLM) at the freshly cut tip of Teflon coated metallic wire was first reported by Tien and Salamon [16]. This technique is based on the interaction of an amphiphatic lipid molecule with a nascent metallic surface. One end of a Teflon-coated stainless-steel metal wire (with 0.1–0.5 mm diameter) is immersed in lipid solution and then, while still immersed, the tip is cut off with a miniature guillotine (Figure 2). The tipped wire, having become coated with lipid is placed in electrolyte solution (usually 0.1 M KCl, mixed with the usual buffers, e.g., Tris HCl or HEPES), whereupon the lipid film spontaneously thins, forming a self-assembled lipid bilayer.

sBLMs have been extensively studied [16,17,18]. The diameter and composition of the wires used were found to play an important role in the time required for device stabilization, and in the magnitude of the background ion current (noise) [17,18]. The use of wires with 0.25 mm diameter should be avoided along with the use of decane as a solvent. Silver wires of 0.5 and 1.0 mm diameter have provided BLMs that were mechanically and electrical stable for over 48 h.

Previous studies have provided a model of a potential profile across sBLMs and have evaluated the structure of the inner lipid layer (facing the silver wire support). It has been suggested that the lipid headgroups bind to the electrode by interactions of oxygen atoms of the phosphate groups of the lipids with silver ions in the metal lattice [19,20]. Note that the BLM does not completely insulate the silver metal from the chloride ions in the electrolyte solution. Furthermore, chloride ions can also readily move through the lipid film during the initial BLM self-assembly process and chloride would react with the silver metal to form silver chloride [17,18]. Interestingly, when the BLM is removed for the silver wire using organic solvent rinse, potentiometric (against a Ag/AgCl reference electrode) experiments [17] show only small voltages (relative to a silver wire against a Ag/AgCl reference electrode). These results suggest that the surface of the metal is likely coated with a thin layer of silver chloride and that the sBLM actually consists from smaller BLMs on the order of nm that provide a nanostructure in these devices [21].

More options are available from the Langmuir-Blodgett (LB) technology that allows lamellar lipid stacking by transferring a monomolecular film formed at the air/water interface onto a solid support. The possibility of building bilayer-based architectures to suit any need is intriguing indeed, provided that the end result is free from disclinations, crystalline or collapsed domains, and later inhomogeneities [22]. Yet, once optimized, this technique might be readily applicable in molecular electronic devices.

Vesicle spreading (fusion) is a less instrument-demanding technique, where rupturing of unilamella vesicles on an oppositely-charged support leads to a bilayer assembly covering the support [23]. Bioelements can be incorporated into the sBLM during vesicle formation, i.e., prior to bilayer formation, or adsorbed onto the prepared bilayer membrane. Both the process of membrane deposition and the physical properties of the membrane strongly depend on ionic interactions between lipids and the support.

### 2.2. BLMs Formed on Glassy Carbon Electrodes

Glassy carbon is inert and has a wide potential window in electrochemistry; these properties render it an appropriate support for the formation of sBLMs. Siontorou et al. [24] have demonstrated this event by successfully incorporating DNA into lipid bilayers supported on the surface of a glassy carbon electrode (GCE). The procedure involved the spreading of single stranded DNA in solution over a pre-formed bilayer; the DNA-membrane interaction has been confirmed with differential pulse voltammetry. Wu et al. [25] used a similar approach: they formed sBLMs by spreading a drop (ca. 5 μL) of dimyristoylphosphatidylcholine (DMPC) in chloroform (2 mg/mL) onto the polished surface of GCE that was immediately immersed in phosphate buffer (PBS). The formation of sBLM was verified by impedance spectroscopy. The specific capacitance obtained was 0.37 μF/cm^2^; this value suggests that the thickness of the hydrophobic part of the BLM is 4.8 nm. Huang et al. [26] used a negatively-charged lipid (dipalmitoylphosphatidylglycerol, DPPG) for the preparation of sBLMs on GCE supports. The specific capacitance, determined, also, by impedance spectroscopy, was 0.26 μF/cm^2^. They used these membranes to study the interaction of the antimicrobial peptide nisin with the lipid bilayer. The results provided show that, at relatively low peptide concentration (less than 150 μmol/L), nisin induced the formation of pores in the sBLM that enhanced its conductivity. At higher concentrations (above 750 μmol/L), nisin disrupted the lipid layer acting like surfactant.

Recently, Zhang et al. [27] used a similar approach to support a didodecyldimethylammonium bromide bilayer GCE. The authors studied the ion channel behavior of the supported BLM by scanning electrochemical microscopy using perchlorate anions as stimulants and ruthenium (II) complex cations as the probing ions. The rates of electron transfer reaction in the lipid membranes surface were detected and found dependent on the potential provided.

### 2.3. Stabilized Lipid Films Formed on a Glass Fiber Filter

**Figure 3:**
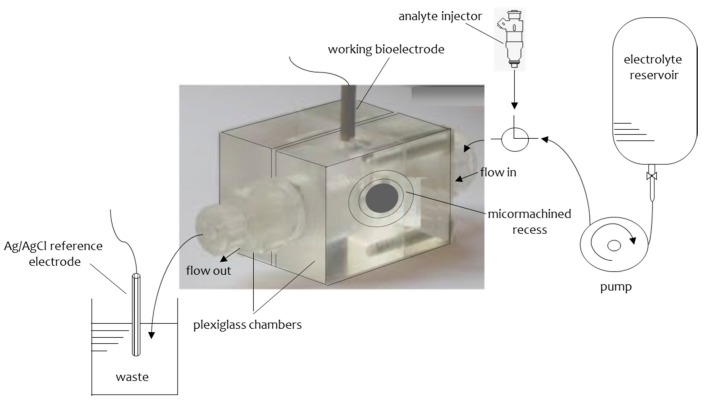
Schematic of the experimental set-up; the micromachined chambers are separated by a thin (12.5 μm thick) polyvinylidene chloride wrap and enclose the microfiber disk. For more details, see text.

Another form of stabilized lipid films that allowed practical implementation were based on ultrafiltration membranes [28]. These supported lipid films proved rugged enough to allow the analysis of real samples, e.g., for the determination of aflatoxin M1 in milk and dairy products [29]. The lipid membrane was formed on a microporous filter glass fiber disk of 0.7 μm nominal pore size [28,29]. The experimental set up used consisted of two plexiglas chambers separated by a Saran Wrap partition of ca. 10 μm thickness; the microfiber disk was mounted around a 0.32 mm orifice machined in the center of the plastic partition (Figure 3). The electrochemical cell and electronic equipment were isolated in a grounded Faraday cage.

The procedure for the formation of the stabilized BLMs was as follows [28,29]: the lipid solution was added dropwise to the electrolyte surface of one chamber; a syringe was used to remove the electrolyte solution from that chamber and refill it again after a few seconds. The formation of the BLMs could be verified by the ion current magnitudes that decreased from μA to a few pA and, confirmed by the electrochemical characterization using gramicidin D. This peptide forms prototypical ion channels that span a bilayer [30]; gramicidin channel gating is known to occur by a well-defined conformational change, i.e., the formation and dissociation of a transmembrane dimer that can occur only at a bilayer thickness [31]. Thus, gramicidin testing is very conclusive for verifying the formation of bilayers, as the channel would not be formed within monolayer or multilayer assemblies.

### 2.4. Polymer Supported BLMs

The process for the preparation of polymerized stabilized BLMs involves either heating the lipid mixture to 60  °C [32,33] or UV irradiation [34,35]. The latter is much preferred as it allows the incorporation of biological moieties in the lipid mixture prior to polymerization [34,35]. The incorporation of the biological element within a forming membrane allows a high degree of conformational changes to occur in order to optimize adsorption; otherwise, a pre-formed membrane allows only surface modifications. As increased temperatures may deactivate many enzymes (i.e., acetylcholinesterase), heat-induced polymerization allowed only for post-membrane formation incorporation.

For the polymerization process, 0.8 mL of a mixture containing 4% *w*/*v* egg phosphatidylcholine (egg PC) in n-hexane were mixed with 0.07 mL of methacrylic acid, 0.8 mL of ethylene glycol dimethacrylate, 8 mg of 2,2’-azobis-(2-methylpropionitrile) and 1.0 mL of acetonitrile [34,35]. The mixture was sparged with nitrogen for about 1 min and sonicated for 30 min. For the preparation of the stabilized lipid films, 0.15 mL of this mixture were spread on a microfilter (microporous glass GF/F microfiber disk with a diameter of ca. 0.9 cm and nominal pore size of 0.7 µm). The filter with the mixture was then irradiated using the UV deuterium lamp. Raman spectrometry and differential scanning calorimetry (DSC) w used to monitor the kinetics of the polymerization process. The results indicated that polymerization is completed within 4 h. The measuring set up was similar to that presented on Figure 3. The membranes produced were stable in storage in air for repetitive uses.

### 2.5. Lipid Films Supported on Nanomaterials

Graphene nanomaterials received tremendous attention in the field of basic research and in technological applications due to their unique physicochemical properties, i.e., good sensing ability, and excellent mechanical, thermal and electrical properties. ZnO nanomaterials also had attracted considerable interest in the field of sensors due to similar advantages, which include large surface-to-volume ratio, excellent biocompatibility, high electron-transfer rates, non-toxicity, and bio-safety. Their implementation in electrochemical biosensing is quite beneficial as the large surface-area-to-volume ratio enables miniaturization, increases speed of response and allows for lower detectabilities while solving the biocompatibility and biofouling problems. Several examples in the development of nanobiosensors by integrating enzymes and antibodies were recently described in literature. Stabilized lipid films were wrapped around a copper wire containing graphene nanosheets [36,37] or ZnO microelectrodes [38]. These nanosensors have been implemented in the rapid detection of environmental pollutants and toxins in real samples, such insecticides [37], naphthalene acetic acid [39], cholera toxin [40], and saxitoxin [41].

The preparation of graphene microelectrodes was as follows [36,37,38,39,40,41]: a homogeneous graphene dispersion (ca. 0.4 mg/mL) has been obtained in N-methyl-pyrrolidone (NMP) through mild sonication for 180 h and centrifugation at 700 rpm for 2 h. This suspension has been poured onto a copper wire (of 0.25 mm diameter) mounted on a glass fiber filter and evaporation of the organic solvent has been carried out using a fan heater. This copper wire has been utilized to establish the connection for the extraction of voltage signals for the calibration curve. Thus, a simplistic approach of drop wise dispersion of graphene suspended in NMP solution has been utilized to scatter the graphene nanosheets on the copper wire. The extended sonication time results in a good fraction of monolayer sheets but with smaller lateral sizes.

Stabilized lipid films were prepared by UV-induced polymerization with a procedure similar to that previously described [34,35], using a different lipid mixture. Briefly, 0.15 mL of a mixture containing 5 mg of a mixed lipid powder composed of 35% (*w*/*w*) dipalmitoyl phosphatidic acid (DPPA), and 65% (*w*/*w*) of dipalmitoyl phosphatidyl choline (DPPC), i.e., 1.75 mg DPPA and 3.25 mg DPPC, were mixed with 0.070 mL of methacrylic acid, 0.8 mL of ethylene glycol dimethacrylate, 8 mg of 2,2’-azobis-(2-methylpropionitrile), and 1.0 mL of acetonitrile. The biological moieties, i.e., enzymes, antibodies, or receptors, were incorporated in these BLMs prior to polymerization by spreading 15 µL of the bioelement suspension with the polymerization mixture (i.e., for the preparation of the stabilized lipid films, 0.15 mL of the polymerization mixture and 15 µL of bioelement suspension were spread on the microfilter). The preparation of the potentiometric biosensor has been finalized by encapsulation of the filter-supported polymerized lipid film onto the copper wire containing graphene nanosheets.

### 2.6. Micro- and Nano-Fabricated Lipid Bilayers

Advances in lithography allowed the use of microfabricated devices for bilayer construction. Two approaches are available: (i) preparation of lipid bilayers in micro-apertures, and (ii) automatic formation of lipid bilayers in microfluidic devices. Most of the apertures fabricated are vertically orientated (instead of laterally) resulting in the formation of bilayers in a horizontal plane [42]. Silicon or other hydrophobic material can be used for the fabrication of the apertures. Decreasing the size of the aperture, decreases electrical noise, as well; however, apertures less than 40 μm allow external noise to become dominant.

Automatic formation of solvent-containing lipid bilayers in microfluidic devices was proposed by (i) solvent extraction through the walls of a microfluidic channels, (ii) assembling two lipid monolayers at the interface between organic and aqueous phases, and (iii) flowing lipid solution and aqueous buffer alternately into a microfluidic channel [43]. Simultaneous parallel recordings of alamethicin and gramicidin have been demonstrated [44], showing a significant potential for multi-channel monitoring.

A new trend in micropatterning biological arrays focuses on the use of membrane-mimicking materials that are more susceptible to surface engineering. Parylene, a family of chemically vapor-deposited polymer, has received much attention lately, especially as regards patterning of biomolecules in hydrated environments [45]. Combined with high resolution imaging techniques, subcellular physical processes can be readily monitored [46]. Parylene has been also suggested as lipid membrane support with excellent stability [47]. Although parylene-based membrane patch fabrication and sensing have been currently implemented in biomedical applications for the development of wireless implantable sensors [48], their adoption to environmental sensing is expected to provide several advantages for reliable quality assessments.

## 3. Applications of Lipid Membrane Nanosensors for Environmental Monitoring

A number of papers that have appeared in the literature have provided devices for the rapid detection of environmental pollutants based on lipid film technology and nanobiosensing. These papers include nanobiosensors for the rapid detection of insecticides, pesticides, hydrazines, naphthalene acetic acid, arochlor, toxins, polyaromatic hydrocarbons, etc. The main characteristics of the sensors described below are summarized in Table 1. The devices included in this review have been selected on the basis of adequate analytical validation data, especially with respect to selectivity for the target analyte and interference studies. Adequate data on operational stability has been published with respect to the polymerized membrane platforms. Some devices have been implemented in real sample analysis, but only one has been implemented at the pilot scale so far. In all cases, samples are minimally processed for extracting the analyte and used without pretreatment or conditioning.

A miniaturized potentiometric carbofuran chemical sensor on graphene nanosheets with incorporated lipid films has been described in the literature [37]. The graphene electrode was used for the development of a selective and sensitive chemical nanosensor for the detection of carbofuran by immobilizing an artificial selective receptor (calix[4]arene phosphoryl receptor) on the stabilized lipid films. This chemical sensor could detect carbofuran concentrations at nM concentration levels, with fast response times of ca. 20 s, simple sensor assembly process and good reproducibility, reusability, selectivity, long shelf life, and high sensitivity of ca. 59 mV/decade over the carbofuran logarithmic concentration range from 10^−6^ to 10^−3^ M.

A work that investigates the interactions of atrazine with bilayer lipid membranes (BLMs) that can be used for the direct electrochemical sensing of this herbicide has been also reported [49]. The interactions of atrazine with solventless BLMs were found to be electrochemically transduced by these membranes in the form of a transient current signal with a duration of seconds, which reproducibly appeared within 1 min after exposure of the membranes to atrazine. The sensitivity of the response was maximized when the lipid composition was enriched with 35% (*w*/*w*) DPPA and the bulk solution with calcium ions that altered the phase distribution within the membrane. The mechanism of signal generation was related to the adsorption of atrazine with a consequent rapid reorganization of the membrane electrostatics due to atrazine aggregation at the surface of BLMs. Hydrogen bonding between atrazine and the carbonyl group of the lipid was explored by addition of platelet-activating factor (PAF; an ether analog of PC) in BLMs composed of phosphatidyl choline. The aggregation of atrazine in membrane domains enriched in the charged lipid have been studied with differential scanning calorimetry of vesicles composed of 15% DPPA. The magnitude of the transient current signal was linearly related to the concentration of atrazine in bulk solution with sub-micromolar detection limits. This electrochemical transduction of atrazine interactions with BLMs holds prospects for flow injection monitoring of triazine herbicides.

The above concept has been implemented in a filter-supported bilayer lipid membranes (BLMs) system for the simultaneous detection of simazine, atrazine, and propazine in mixtures [50]. The time of appearance of the transient signal was different for each triazine and increased to the order of simazine, atrazine and propazine which has allowed their selective detection and analysis in the same sample. In this case, the lipid bilayer alone served as the biorecognition element (as described in panel (iii) of Figure 1). The mechanism of signal generation involved a two-step process: triazine molecules adsorbed to the surface of the BLM associate as aggregates to provide electrostatic perturbation of the lipid membrane [55]. The rate of aggregation is slow as it is based on the movement of triazines across the surface of the membranes. As the triazine herbicides become bulkier in the order of simazine, atrazine (plus one methyl group), and propazine (plus two methyl groups), the steric hindrance provided reduces the rate of aggregation at the same order. This was demonstrated as differences in the time of appearance of the signal related to each herbicide: 34–50 s for simazine, 62–78 s for atrazine, and 96–144 s for propazine [50].

A similar approach using metal supported lipid membranes (s-BLM) has been also suggested by [18]. The sensor, based on bilayers self-assembled on the electrode tip, did not exert any discrimination capability and provided a cumulative response for the three triazines. As evident, the metal-adherence architecture and the chemistry within the lipid-metal interface limit the degrees of freedom for membrane reconfiguration during the biochemical interactions; further, surface confinement might hinder aggregation kinetics.

A paper has appeared in the literature that describes a strategy for the electrochemical monitoring of changes of ion current through a lipid membrane with immobilized DNA probes caused by interaction of these modified BLMs with hydrazine compounds [51]. A self-assembled metal supported bilayer lipid membrane (s-BLM) composed of egg PC was prepared on a silver metal electrode. The oligomers used were single stranded deoxyribonucleic acids: thymidylic acid icosanucleotide terminated with a C-16 alkyl chain to assist incorporation into s-BLMs (dT20-C16), and deoxyadenylic acid icosanucleotide (dA20). These s-BLMs with incorporated DNA interacted with hydrazines, and the s-BLMs displayed an analytically useful tool for ppb detection levels of hydrazine compounds (i.e., hydrazine, methylhydrazine, dimethylhydrazine, and phenylhydrazine). This BLM/DNA biosensor offers a highly sensitive, rapid, and portable device for monitoring these environmentally- and toxicologically-significant compounds.

A miniaturized potentiometric naphthalene acetic acid (NAA) nanosensor on graphene nanosheets with incorporated lipid films has been recently described [39]. A receptor (auxin-binding protein 1 receptor) was immobilized on the stabilized lipid films on graphene electrodes. The proposed sensor has provided adequate selectivity, sensitivity (mM detection levels), fast response time (ca. 5 min), is easy to construct, and exhibits good reproducibility, reusability, long shelf life, and a sensitivity slope of ca. 56 mV/decade of hormone concentration. The reliability of the biosensor was successfully evaluated using a wide range of NAA-spiked fruits and vegetables.

The potentiometric detection of cholera toxin has been also suggested using graphene nanosheets with incorporated lipid films [40]. Ganglioside GM1 (a natural cholera toxin receptor) was immobilized on stabilized lipid films on graphene electrodes, provided adequate selectivity for detection over a wide range of toxin concentrations, fast response time of ca. 5 min, and detection limit of 1 nM. The proposed sensor is easy to construct and exhibits good reproducibility, reusability, selectivity, long shelf life, and a slope of ca. 60 mV/decade of toxin concentration. The method was implemented and validated in lake water samples. This novel ultrathin film technology is currently adapted to the rapid detection of other toxins and could be used as a weapon against bioterrorism.

A novel electrochemical biosensor based on a supported polymerized lipid films with incorporated sheep anti-PCB antibody for the rapid detection of arochlor 1242 in flowing solution streams has appeared in the literature [52]. The antibody was incorporated into the lipid film during polymerization. Additionally, injections of antigen were made into flowing streams of a carrier electrolyte solution. The experiments were performed in a stopped-flow mode using lipid mixtures containing 15 % (*w*/*w*) DPPA to provide only a single transient current signal with a magnitude related to the antigen concentration. Lipid films containing 35 % DPPA were used to examine the regeneration of the active sites of antibody after complex formation by washing with the carrier electrolyte solution. Repetitive cycles of injection of antigen have shown that the maximum number of cycles was ca. 5.

A miniaturized potentiometric saxitoxin sensor on graphene nanosheets with incorporated lipid films and Anti-STX (the natural saxitoxin receptor) immobilized on the stabilized lipid films was described recently in a paper [41]. A good selectivity for the detection over a wide range of toxin concentrations, rapid response times of ca. 5–20 min, and detection limits of 1 nM were achieved. The proposed sensor was easy to construct and exhibited good reproducibility, reusability, selectivity, long shelf life, and a sensitivity of ca. 60 mV/decade over the toxin concentration. The method was implemented and evaluated in lake water and shellfish samples. This novel ultrathin film technology could be easily adapted to the rapid detection of other toxins that could be used as weapons against bioterrorism.

Shiratori et al. [53], have recently proposed a novel gas sensor based on LB films and fullerenes as spacers. The spacers were used to control the lipid bilayer deposition on a quartz crystal microbalance (QCM) electrode. The permeability of gaseous polyaromatic hydrocarbons into the film was found to be increasing as molecular weight or molecular size decreased. This molecular sifter function has been attributed to the interaction of the gas molecules with lipid bilayer.

In recent years, Ishimori et al. [54] have developed an advanced environmental monitoring system based on lipid membrane sensors for the continuous monitoring of underground water. The system links the sensors with a computer-based pollutant propagation simulation. An automatic BLMs preparation device was made based on an inkjet mechanism. The sensitivity to volatile organic chloride compounds such as cis-1,2-dichloroethylene was in the order of 10 ppb using the monoolein BLMs to analyze real underground water samples. The system is currently implemented at the pilot scale.

Optical transduction strategies coupled to lipid bilayers have not been commonly suggested for environmental applications. Yet they have been commonly employed in monitoring protein-lipid interactions (e.g., see [14]) and ion channel monitoring (e.g., see [56,57,58,59] for some applications), as well as in food analysis (e.g., see [60,61]). Their adoption in environmental analysis might provide certain benefits, especially as regards the monitoring of the degradation of pollutants in situ.

Despite the advancements witnessed in the field of lipid membrane based biosensors, moving from solventless BLMs to rugged polymerized lipid films and metal-supported constructs, the development of in the field devices presents certain challenges that still need to be addressed. A critical issue derives from the sample itself. Environmental samples are complexes containing numerous compounds at various states and the engineer can only work with rough estimates of anticipated matrix effects. In the field sensor operation should also consider variations (sometimes abrupt) in both, environmental parameters (such as temperature) and analyte concentration levels. Currently, sensor design follows a more or less analyte-based approach: the analyte is paired with a bioelement and the nature of their interaction influences the selection of the transduction strategy; if selectivity can be enhanced by adjusting macro-parameters (pH, ionic strength, etc.), a proof-of-concept is reported. Although simplistic enough, this description highlights that the environment under which the sensor is intended to operate is rarely considered at the early development phases. On the other hand, the inclusion of environmental parameters in sensor design with a view to producing devices with built-in ruggedness requires in-depth knowledge of the monitoring site [62]. Apart from the obvious problems with the availability of this knowledge, different sites might differ in many aspects and, thus, in the field sensors should adopt a more site-specific development approach based on versatile platforms, easily engineered in ‘as needed’ systems.

## 4. Conclusions and Future Prospects

The present paper describes the various routes for the preparation of nanosensors based on a lipid film technology for environmental analytical applications. Recent technological advances include the construction of stabilized supported lipid film on graphene nanoelectrodes with an incorporated “receptor” (enzyme, antibody, or natural or artificial receptor) stable for storage in air that can be portable for field applications. These sensors reveal detection limits in the nanomolar range. The most important aspect of the present efforts is to provide a commercial portable device that can be used for in situ applications.

The results have shown that a diversity of lipid film based sensing platforms can be reused after storage in air even for a period of a couple of months. These sensors can be reproducibly fabricated with simplicity and low cost to offer rapid response times, operational stability, and care-free real sample analyses. Compared to chromatography and antibody-based environmental detection methods, biosensors can serve as complementary in-field gauzes.

Recently-advanced lithography will enable the fabrication of nanopores and the insertion of nanoparticles in thin membranes where artificial bilayers can be assembled. This process accommodates a wide range of lipid composition in stable form, with the inclusion of membrane proteins. Therefore, the application of nanotechnology to this field of lipid film technology will allow miniaturization and will result in the mass production of sensors. Producing the smaller patterns will enable sensors to respond faster, with a higher degree of sensitivity, and at lower production costs. Development of sensors using the present technologies will offer improved sensitivity for detection with high specificity at the molecular level, with an increment of several order of magnitude over currently available techniques with a large number of applications in environmental monitoring.

## Figures and Tables

**Table 1:** Overview of lipid membrane nanosensors developed for environment monitoring.

Pollutant Class	Bioelement	Membrane System/Detection Method	Analytical Performance	Real Sample Analysis	Reference
carbamate pesticides: Carbofuran	calix[4]arene phosphoryl receptor	graphene nanosheets with incorporated lipid films/potentiometric	RT: 20 s	fruits and vegetables	[37]
			DL: 100 nM		
triazine herbicides: Atrazine	N/A	solventless BLMs doped with dipalmitoyl phosphatidic acid and platelet activating factor/electrochemical	RT: 55.6 ± 5.4 s	N/A	[49]
			DL: 0.1 ppm		
triazine herbicides: Simazine; Atrazine; Propazine	N/A	Filter supported BLMs doped with dipalmitoyl phosphatidic acid/electrochemical	RT: 34–50 s (simazine); 62–78 s (atrazine); 96–144 s (propazine)	N/A	[50]
			DL: 18 ppb (simazine); 0.05 ppm (atrazine); 30 ppb (propazine)		
triazine herbicides: Simazine; Atrazine; Propazine	N/A	Metal supported BLMs doped with dipalmitoyl phosphatidic acid/electrochemical	RT: 10 s	N/A	[18]
			DL: 1 ppb (simazine); 15 ppb (atrazine); 30 ppb (propazine)		
hydrazines: Hydrazine; Methylhydrazine; Dimethylhydrazine; Phenylhydrazine	N/A	Metal supported BLMs doped with ssDNA/electrochemical	RT: 18–20 s	N/A	[51]
			DL: 51.5 ppb (hydrazine); 0.005 ppb (methyl hydrazine); 0.02 ppb (dimethylhydrazine); 0.11 ppb (phenylhydrazine)		
plant growth regulators: Naphthalene Acetic Acid	auxin-binding protein 1 receptor	graphene nanosheets with incorporated lipid films/potentiometric	RT: 5 min	fruits and vegetables	[39]
			DL: 10 nM		
toxins: Cholera toxin	ganglioside GM1	graphene nanosheets with incorporated lipid films/potentiometric	RT: 5 min	lake water samples	[40]
			DL: 1 nM		
polychlorinated biphenyls: Arochlor 1242	sheep anti-PCB antibody	Filter supported polymerized lipid films doped with dipalmitoyl phosphatidic acid/electrochemical	RT: 45–55 s	N/A	[52]
			DL: 10 nM		
toxins: Saxitoxin	anti-STX receptor	graphene nanosheets with incorporated lipid films/potentiomeric	RT: 5–20 min	shellfish samples and lake water samples	[41]
			DL: 1 nM		
polyaromatic hydrocarbons	N/A	Fullurene spatially controlled LB fims/quartz crystal microbalance	*Not reported*	indoor air	[53]
volatile organic chlorides	N/A	Inkjet printed BLM from lipid droplets/electrochemical	RT: 1 min	underground water samples	[54]
			DL: 10 ppb		

RT: response time; DL: detection limit.
